# Diabolical dilemmas of COVID-19: An empirical study into Dutch society’s trade-offs between health impacts and other effects of the lockdown

**DOI:** 10.1371/journal.pone.0238683

**Published:** 2020-09-16

**Authors:** Caspar Chorus, Erlend Dancke Sandorf, Niek Mouter

**Affiliations:** 1 Department of Engineering Systems and Services, Faculty of Technology, Policy and Management, Delft University of Technology, Delft, Netherlands; 2 Economics Division, Stirling Management School, University of Stirling, Stirling, Scotland; University of York, UNITED KINGDOM

## Abstract

We report and interpret preferences of a sample of the Dutch adult population for different strategies to end the so-called ‘intelligent lockdown’ which their government had put in place in response to the COVID-19 pandemic. Using a discrete choice experiment, we invited participants to make a series of choices between policy scenarios aimed at relaxing the lockdown, which were specified not in terms of their nature (e.g. whether or not to allow schools to re-open) but in terms of their effects along seven dimensions. These included health-related effects, but also impacts on the economy, education, and personal income. From the observed choices, we were able to infer the implicit trade-offs made by the Dutch between these policy effects. For example, we find that the average citizen, in order to avoid one fatality directly or indirectly related to COVID-19, is willing to accept a lasting lag in the educational performance of 18 children, or a lasting (>3 years) and substantial (>15%) reduction in net income of 77 households. We explore heterogeneity across individuals in terms of these trade-offs by means of latent class analysis. Our results suggest that most citizens are willing to trade-off health-related and other effects of the lockdown, implying a consequentialist ethical perspective. Somewhat surprisingly, we find that the elderly, known to be at relatively high risk of being affected by the virus, are relatively reluctant to sacrifice economic pain and educational disadvantages for the younger generation, to avoid fatalities. We also identify a so-called taboo trade-off aversion amongst a substantial share of our sample, being an aversion to accept morally problematic policies that simultaneously imply higher fatality numbers and lower taxes. We explain various ways in which our results can be of value to policy makers in the context of the COVID-19 and future pandemics.

## 1. Introduction

The outbreak of COVID-19 in the Netherlands, as in many other countries, was followed by an unprecedented package of measures, summarized by the Dutch government under the name "intelligent lockdown". As of mid-March, schools closed, as did bars and restaurants and countless other service providers (the so-called 'contact professions' such as barbers). Working from home became the norm, large scale events such as professional football matches were banned, and a variety of other commandments and urgent stipulations were issued [1].

In this first, acute phase of the crisis, there was a sense of unanimity and focus in Dutch society: the goals (i.e., delaying and limiting the spread of COVID-19, protecting the vulnerable, preventing the collapse of the healthcare system) justified the means (i.e., a lockdown that in effect crippled large parts of society). In this acute and early phase of the crisis, deontological ethics were dominant in the public debate: most agreed that everything had to be done to keep the healthcare sector afloat and to keep the loss of human lives at an absolute minimum.

About a month and a half after the lockdown was put in place, pressure on the healthcare system started to gradually decrease, accompanied by a downward trend in the number of COVID-19 related fatalities. This signaled a gradual transition into the chronic phase of the crisis, with public attention shifting to the question of how the country should deal with the lurking threat of a virus that can always re-emerge until a vaccine or medicine is found, while at the same time keeping society functioning at a reasonable level.

In the public debate it is becoming clear that this chronic phase also entails a different–consequentialist–ethical perspective in which all possible effects of government policy are taken into account. In addition to health related effects, this includes, for example, the impacts of the lockdown on the economy at large and one’s personal income, as well as possible educational disadvantages due to distance learning. During this transition to the chronic phase of the crisis, the call for further opening up society has been getting louder and louder. The Dutch government–like many other ones–as a result found itself in a position where diabolic dilemmas had to be faced; these were the words uttered by Prime Minister Mark Rutte, during an April 21 press conference watched by almost half of the Dutch population [2].

The morning after this press conference, our survey including a choice experiment went live. Our aim was to explore the preferences of the Dutch population in terms of the weights they assigned to various effects of policies aimed at relaxing the lockdown. Specifically, we wanted to know if the Dutch were willing to trade health effects (such as avoiding fatalities) against other effects (e.g. on the economy), and if so, what would be their willingness to sacrifice economy- and education-related suffering for a reduction in fatalities and in pressure on the national healthcare system be. Their choices in the experiment would also allow us to learn to what extent the transition from a deontological ethics perspective (requesting a full focus on saving human lives) was indeed gradually being replaced by a consequentialist one which puts weight on all foreseeable consequences of government policy.

This paper reports the outcomes of this choice experiment. To be sure, the main contribution of this paper is not a methodological one: the use of choice experiments to study citizens’ preferences and trade-offs involving fatalities and injuries has a long tradition in health economics [3], traffic safety analysis [4] and environmental and climate change economics [5]. More related to the topic of our study, choice experiments have been deployed for measuring preferences for a COVID-19 contact tracing app in the Netherlands [6,7], in the United Kingdom [8] and in the United States (e.g. [9]); as well as to measure preferences for attributes of a COVID-19 vaccine among Australian citizens [10]. In addition, related survey techniques have been used to study beliefs about the effectiveness of COVID-19 policies [11], public sentiment toward COVID-19 policies [12] and willingness to be vaccinated against COVID- 19 [13]. What makes our study unique is that it is the first one, to the best of our knowledge, that applies this tool to survey and investigate a society’s willingness to make the highly salient and morally troubling trade-offs associated with policies aimed at relaxing a lockdown that was imposed in the wake of the COVID-19 crisis.

While our study is confined to the Dutch society, and we acknowledge that countries differ widely in terms of their culture, the actions taken by their governments and preferences towards COVID-19 policies [11,12], we nonetheless observe that in many other countries, debates are raging that are similar to the one being held in the Netherlands; take for example the heated exchange in the United States of America (e.g., [14]) between governor Cuomo of New York who emphasizes that avoiding fatalities takes priority and that one cannot weigh a human live against the economic impact of the lockdown, versus president Trump who is keen to re-open the economy and professes that the cure cannot be worse than the disease. Given the similarities between various countries in terms of the societal tension between health- and economy-related effects of lockdowns, we expect our results to hold lessons well beyond the Dutch context.

The remainder of this paper is organized as follows: section 2 presents the method of discrete choice experiments and the econometric methodology used to analyze the obtained choice data. Section 3 elaborates the data collection effort, followed by section 4 where we present and interpret our results. Section 5 concludes by discussing the main conclusions and policy recommendations that can be drawn from our study.

## 2. Methods

The method of using discrete choice experiments (DCEs) to elicit latent preferences and trade-offs of citizens regarding the effects of government policies has a long pedigree in domains as diverse as transport (e.g. [15]), environment and climate adaptation [16,17], immigration [18] and health care [19–21]. Also when it comes to morally challenging trade-offs involving human lives, DCEs have been widely used in these contexts (e.g. [22,23]). The core idea behind using DCEs is that choices made by respondents between policy scenarios specified in terms of their outcomes on various dimensions, can be used to identify the weights that respondents assign to each of these dimensions. These weights may then be used to: i) learn about the relative importance attached by society to various policy-impact dimensions, ii) predict levels of support for and opposition against specific policies, and iii) convert various policy dimensions into monetary terms in order to allow for a cost-benefit assessment. In this paper, the emphasis is on the first of these three potential uses of DCEs.

Compared to other approaches to identify citizens’ preferences and trade-offs, such as directly asking respondents to assign a weight to various policy-impact dimensions, the DCE methodology has important advantages. For example, it is well known that people find it very difficult to make explicit their preferences and decision making processes [24], especially in the context of morally sensitive topics such as the one we study [25]. That is, people have great difficulty in reliably answering questions such as “how many cases of lasting mental health effects are you willing to tolerate, to avoid one COVID-19 related fatality?”. The DCE approach circumvents such difficulties, by asking respondents to choose between policy scenarios specified in terms of these and other impacts; based on choices made, the implied relative weights attached to different dimensions can then be inferred by the analyst. Furthermore, in contrast to standard opinion polls which typically ask questions that are too generic to be of much policy relevance (“should lockdown policies be focused more on reducing health effects or economic effects?”), the DCE approach presents very specific policy scenarios (e.g. in terms of numerically expressed policy effects), allowing for the assessment of particular policies based on the estimated weights.

It is important to note however, that despite its advantages and the resulting widespread use of DCEs, there is a continuing debate about their reliability [26,27]. Important insights from this debate can be put as follows: i) if available, the analysis of choices observed in real life (e.g. during referenda) is to be preferred over the analysis of choices made in hypothetical conditions such as a DCE; ii) if a DCE is used, care must be exercised to ensure ‘consequentiality’, i.e. respondents must feel that their choices will have consequences in real life [28]; iii) the choice situations presented in the DCE must be realistic and must align with experiences and considerations held by respondents (see e.g. [29,30]). A recent study in the context of immigration policies in Switzerland, which compared the outcomes of a DCE with those obtained by an actual referendum, shows that a properly designed DCE is likely to generate reliable insights into the weights assigned by the population to various policy dimensions, also in morally salient contexts [31].

Translating these generic lessons to our specific DCE, we are confident that the policy scenarios presented to respondents were well aligned with the current public debate in the Netherlands; in fact, the dimensions covered in our study were widely discussed in the media during the weeks preceding the data collection. Furthermore, we made a substantial effort to ensure consequentiality, by (truthfully) informing respondents that the outcomes of this study would be shared with high-ranking policy makers at relevant Ministries and the Netherlands Institute of Public Health (RIVM). Qualitative statements provided by respondents after having completed our survey (not reported here) strengthen our belief that most took the experiment very seriously. In the absence of an actual referendum on the topic, we believe that given these precautions our DCE provides a useful and reliable alternative to collect choice data.

In our DCE, we vary policy scenarios along seven dimensions, covering those impacts of the lockdown that received the most attention in the public debate during the weeks preceding the data collection effort. Table 1 shows the different policy dimensions (‘attributes’) and the ranges of their scores (‘attribute levels’).

**Table 1:** An overview of the attributes (policy impacts) and their levels.

Attribute	Level 0	Level 1	Level 2	Level 3
Increase in number of deaths caused by the corona crisis directly or indirectly (e.g. due to postponed operations)^†^	8 000	11 500	15 000	18 500
Increase in number of people with lasting physical injuries caused by the corona crisis directly or indirectly (e.g. due to postponed operations)^†^	30 000	80 000	130 000	180 000
Increase in number of people with lasting mental injuries caused by the corona crisis^†^	20 000	80 000	140 000	200 000
Increase in number of children with lasting educational disadvantages caused by the corona crisis^†^	10 000	90 000	170 000	250 000
Increase in number of households with net income loss of more than 15% for a period of more than 3 years caused by the corona crisis	400 000	700 000	1 000 000	1 300 000
One-off corona tax per household in 2023	€1 000	€2 500	€4 000	€5 500
Work pressure in the health sector during the period May 1st 2020—January 1st 2021	Same work pressure as before the coronavirus crisis	Work pressure lies between the current situation and the situation before the coronavirus crisis	Work pressure is the same as in the current situation	Work pressure is higher than in the current situation

^†^Respondents were informed that while these changes occur during the period May 1st 2020—January 1st 2021, their effects are lasting.

Note that these attributes and their levels were selected in an iterative process of pilot testing and discussions with colleagues at other Dutch universities as well as analysts at relevant Ministries and the Netherlands Institute of Public Health (RIVM). For instance, we decided in consultation with analysts from the Dutch government to select the three health dimensions (‘increase in the number of deaths caused by the coronavirus’, ‘increase in the number of lasting physical injuries caused by the coronavirus’, ‘increase in the number of lasting mental injuries caused by the coronavirus’) instead of using the concept of Quality Adjusted Life Years (QALY) which is popular in health economics studies [32,33]. The reason being that the public debate in the Netherlands focused on these three dimensions and not on QALYs. Moreover, in a draft version of the DCE we made a distinction between increases in the number of deaths in different age groups (younger than 50 years, 50–75 years, older than 75 years); however, policy makers and analysts from the RIVM argued that this was not a relevant variable for the trade-offs they faced in their decision-making. Hence, we decided not to distinguish between different age-groups in our final experiment.

More generally speaking, we constructed the attribute levels in three stages. Firstly, we analyzed studies which provided projections of the impacts of the corona crisis on the seven policy impact dimensions. For instance, we analyzed rough estimates on the increase in the number of deaths [34], projections regarding the increase in the number of people with lasting physical injuries caused by postponed operations [35], data on the increase in domestic violence caused by the corona crisis in the United Kingdom [36], data regarding domestic violence in the Netherlands prior to the corona crisis [37], data on the number of children with educational disadvantages prior to the crisis [38] and projections about bankruptcies, unemployment and income loss [39–41]. Secondly, we discussed the realism of these figures with epidemiologists and policy makers. The epidemiologists warned us to lower our predictions on the number of deaths and gave suggestions for determining the levels for ‘number of people with lasting physical injuries’. Moreover, we asked policy makers from the Ministry of Finance to provide a prediction of the one-off corona tax per household in 2023. Thirdly, we tested in a pilot survey whether the levels that we constructed were salient and relevant in the eyes of participants. Based on the results of the pilot survey we decided to increase the difference between the levels of ‘increase in number of deaths’.

We chose to execute a so-called unlabeled DCE, which did not specify policies in terms of their nature (e.g. reopening schools, or sport clubs) but rather focused on the impact of policies on a range of dimensions. The advantage of such an unlabeled approach is that it allows policy makers to use our results for the assessment of (combinations of policies), including those that are currently not on the table but might be considered in later phases of the crisis.

**Fig 1:**
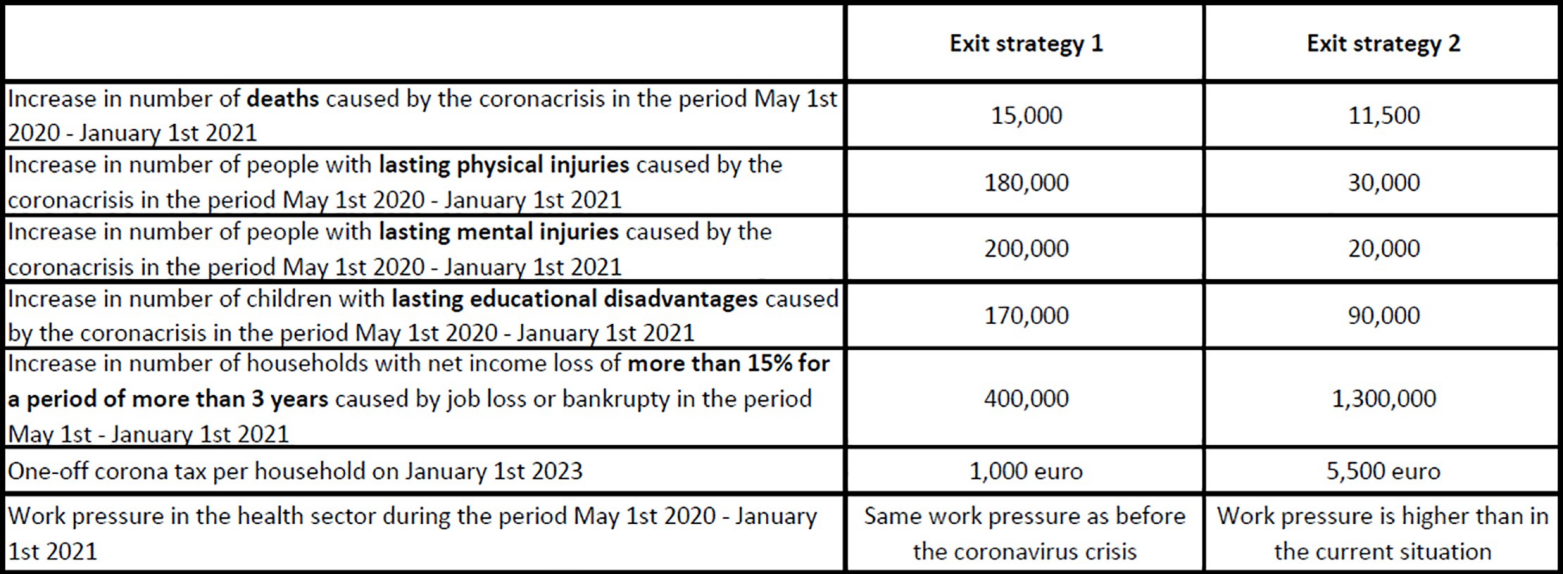
A choice task as presented to respondents.

The experiment was designed using statistical techniques which ensure that every choice task contains a maximum amount of information on the weights assigned by respondents to different policy impacts [42]. More specifically, respondents were asked to choose between two policy packages described by seven attributes or policy impacts. The attributes and their levels (note that each attribute had three possible levels) were combined into 18 choice tasks using a d-efficient design with priors obtained from a pilot study [43]. The 18 choice tasks were blocked into two blocks of 9 choice tasks and respondents were randomly allocated to a block when entering the survey. We show a sample choice task and the text shown to respondents in Fig 1. To see the relation between the Exit strategies presented in the choice task exhibited in Fig 1 with the attribute levels presented in Table 1, note that the left-hand side strategy in the choice task (“Exit strategy 1”) consists of level 2 of attribute Deaths, level 3 of attribute Injuries, level 3 of attribute Mental injuries, level 2 of attribute Educational disadvantages, level 0 of attribute Income losses, level 0 of attribute corona tax, and level 0 of attribute Work pressure in health sector.

Our econometric approach for analyzing the choices collected in the discrete choice experiment involves three model types. First, we estimate a classical linear in parameters binary logit model, where each attribute is assigned a corresponding parameter (its weight) in a process of maximum likelihood estimation; see Ben-Akiva and Lerman [44] for details of this model and how it is estimated. Second, we estimate a latent class model where we identify different classes in the population with people assigned to the same class have the same weight-parameter, i.e., have the same preferences [45,46]. Third, acknowledging the moral salience of the choice context, we estimate a so-called taboo trade-off aversion model [47]. This model estimates a penalty parameter for each policy scenario that involves higher numbers of fatalities and lower taxes. Such a policy may be considered taboo by some respondents, as it in effect assigns a monetary value to a human life. The taboo trade-off aversion effect has a rich tradition in moral psychology [48]), and in the context of a DCE analysis it can be captured by means of an interaction effect, which indicates a potential dislike for a combination of particular attribute values beyond the separate direct effects of those attributes. Following Chorus et al. [47] we implement the taboo trade-off by constructing a dummy variable which equals 1 if a policy scenario presented in a choice task featured lower taxes and more fatalities than the other scenario that was presented in the same choice task. The accompanying parameter estimate represents the level of taboo trade-off aversion (we estimate such a parameter for each latent class, to explore heterogeneity in terms of taboo trade-off aversion).

To introduce notation, let respondent *n*’s utility from choosing alternative *i* in choice situation *s* be described by the linear-in-the-parameters random utility function in Eq 1.

$$U_{nis}=\beta X_{nis}+\varepsilon_{nis}$$ (1)

Here, **β** is a row-vector of parameters to be estimated, column vector **X**_*nis*_ contains the levels of the attributes of the alternative and *ε*_*nis*_ represents an Extreme Value Type I distributed error term. Under these standard assumptions, the probability that respondent *n* chooses alternative *i* in choice situation *s* can be expressed by the multinomial logit model [49].

$$Pr(i_{ns}|X_{nis})=\frac{exp(\beta X_{nis})}{\sum_{j=1}^{J}exp(\beta X_{njs})}$$ (2)

The multinomial logit model (MNL) is the workhorse in discrete choice analysis, but is limited by its inability to describe unobserved preference heterogeneity and its inability to accommodate for the fact that each respondent made a series of choices (panel effect). A latent class model allows for capturing such unobserved preference heterogeneity and panel effects. Let *π*_*qn*_ be the probability that respondent *n*’s preferences can be described by the *q*^th^ vector (class).

$$\pi_{qn}=\frac{exp(\alpha_{q}+\gamma_{q}Z_{n})}{\sum_{q=1}^{Q}exp(\alpha_{q}+\gamma_{q}Z_{n})}$$ (3)

Here, *α*_*q*_ is a class specific constant, **γ**_*q*_ a vector of parameters to be estimated, and **Z**_*n*_ a vector of respondent specific variables. We set the *Q*th constant and parameter vector to zero for identification. Every class *q* has a unique vector of attribute weights **β**_*q*_ which describes the behavioral preferences of respondents assigned to the specific class. This enables us to take the panel structure of the data into account by allowing parameter weights to vary across classes while at the same time ensuring that they are constant across choices made by the same individual. Now, we can express the probability that respondent *n* chooses alternative *i* in a particular choice task *s* as:

$$Pr(i_{ns}|X_{nis},\pi )=\sum_{q=1}^{Q}\pi_{qn}∙\frac{exp(\beta_{q}X_{nis})}{\sum_{j=1}^{J}exp(\beta_{q}X_{njs})}$$ (4)

Likewise, for example, we can express the choice probability that respondent *n* chooses alternative *i* in every choice task *s* as:

$$Pr(i_{n1}\ldots i_{ns}\ldots i_{nS}|X_{ni1}\ldots X_{nis}\ldots X_{niS},\pi )=\sum_{q=1}^{Q}\pi_{qn}∙\prod_{s=1}^{S}\frac{exp(\beta_{q}X_{nis})}{\sum_{j=1}^{J}exp(\beta_{q}X_{njs})}$$ (5)

Given our linear treatment of attributes, society’s willingness-to-sacrifice a particular attribute (e.g. a certain number of households facing an enduring and substantial reduction in net income) in order to avoid one fatality which is directly or indirectly related to COVID-19 can be calculated straightforwardly: it is given by the ratio between the fatality parameter and the parameter associated with the other policy effect (e.g. the parameter associated with the number of households facing income reductions). In the context of the latent class models, the sample level willingness to sacrifice is the weighted sum of the willingness to sacrifice per latent class, where the weights are the unconditional class probabilities.

## 3. Data

The data were gathered on 22 April, the day following a widely watched press conference by Prime Minister Mark Rutte (21 April) during which he emphasized that most of the lockdown-policies and regulations would remain in place until further notice, while some others were slightly relaxed. In hindsight, the days surrounding this press conference can be considered the height of the public debate in the Netherlands about how to weigh the health-related effects of the lockdown with other societal impacts such as economic standstill, educational problems and mental health crises, among others. Note that early May, after our data were collected, another press conference was held in which the government—quite unexpectedly in the eyes of many—announced a series of lockdown relaxations to take place in the months of May through September (also in this, the Netherlands was not alone, as various other countries were contemplating and deciding for relaxations of lockdowns in this same time period).

Respondents were sampled from the online Kantar Public panel, with a view to be representative with respect to the Dutch population in terms of age, gender and education level. Kantar Public approached members of their panel by e-mail to take part in our online survey. Sampled respondents were informed about the purpose of the study, the methods employed and the specific policy impacts that were part of the choice experiment. Our data collection effort was approved by the Ethics Board of the Delft University of Technology. A total of 1260 respondents of the panel were invited to participate. Of these, 1121 respondents started the survey and we received 1,009 completed and usable surveys (implying a drop-out rate of 10%). For our analysis, we excluded two respondents who stated ‘other’ gender because parameters associated with this socio-demographic variable could not be identified empirically due to insufficient variation in the data. All models are run with these respondents excluded for comparability. Table 2 compares the sociodemographics of our sample with those of the population, and finds a close correspondence in terms of gender and age, but an over-representation of highly educated respondents. In the next section, we will explore what this means in terms of the general applicability of our findings.

**Table 2:** Sample and census comparison for gender, age and educational attainment.

	Sample	Census	Test statistic
**Male**	49.4%	49.5%	Chi.Sq. 0.004 df = 1
**Female**	50.6%	50.5%	
**Age 18–25**^**a**^	11.4%	14.7%	Chi.Sq. 4.2408 df = 5
**Age 26–35**	15.1%	15.3%	
**Age 36–45**	14.8%	14.1%	
**Age 46–55**	19.0%	16.9%	
**Age 56–65**	17.9%	16.3%	
**Age 66–75**	13.5%	13.3%	
**Age 75 and older**	8.3%	9.4%	
**Education low**	16.3%	30.6%	Chi.sq. 119.66 df = 2 ***
**Education med**	37.9%	37.4%	
**Education high**	45.8%	32.0%	

^**a**^Under-representation is because the census category is 15–25 and we could only sample 18 and above. Test statistic does not consider this category.

*** Significant at the 1% level

## 4. Results

Table 3 shows the estimation results of our final model specifications. The binary logit model is our point of departure; note that the outcomes of this base model have been published in a Dutch-language Economics journal [50]. Also note that in order to ensure that all obtained parameters were within the same order of magnitude (which helps guaranteeing parameter stability in latent class models), we rescaled our attributes; see Table 3 for the resulting units. Signs are as expected: people dislike increases in: the number of fatalities related (directly or indirectly) to COVID-19; the number of people with lasting physical and mental injuries; the number of children left with an educational disadvantage; the number of households with persistent income loss; personal income taxes; and work pressure in the health care sector. All parameters are highly significant.

**Table 3:** Results from the estimation of the binary logit and three-class latent class model.

	Binary Logit			Class 1			Class 2			Class 3		
Attribute	Est		S.e.	Est		S.e.	Est		S.e.	Est		S.e.
Deaths (per 10,000)	-0.572	***	0.034	-0.291		0.220	-1.026	***	0.137	-0.568	***	0.059
Physical injuries (per 100,000 cases)	-0.588	***	0.023	-1.160	***	0.158	-1.254	***	0.111	-0.313	***	0.051
Mental injuries (per 100,000 cases)	-0.382	***	0.019	-0.280	***	0.097	-1.098	***	0.109	-0.194	***	0.041
Educational disadvantage (per 100,000 children)	-0.308	***	0.014	-0.526	***	0.072	-0.883	***	0.096	-0.093	***	0.031
Income loss (per 1,000,000 households)	-0.729	***	0.039	-1.005	**	0.429	-1.899	***	0.204	-0.381	***	0.075
Tax increase (per 1,000 Euro)	-0.196	***	0.008	-0.981	***	0.079	-0.234	***	0.030	-0.059	***	0.017
Increase in working pressure (health sector)	-0.206	***	0.012	-0.446	***	0.119	-0.269	***	0.039	-0.189	***	0.020
**Class membership functions**												
Constant				-2.900	***	0.853	-1.838	***	0.506	0		(fixed)
Male				0.000		(fixed)	0.000		(fixed)	0		(fixed)
Female				-0.291		0.210	0.435	*	0.225	0		(fixed)
Education low				0.000		(fixed)	0.000		(fixed)	0		(fixed)
Education med				-0.154		0.285	0.119		0.347	0		(fixed)
Education high				-0.020		0.296	1.048	***	0.341	0		(fixed)
Age 18–25				0.000		(fixed)	0.000		(fixed)	0		(fixed)
Age 26–35				2.130	**	0.830	-0.116		0.418	0		(fixed)
Age 36–45				1.762	**	0.843	0.337		0.397	0		(fixed)
Age 46–55				2.508	***	0.823	0.396		0.403	0		(fixed)
Age 56–65				2.584	***	0.829	0.841	**	0.401	0		(fixed)
Age 66–74				2.465	***	0.847	1.246	***	0.453	0		(fixed)
Age 75 +				1.868	**	0.889	0.677		0.540	0		(fixed)
**Average class probability**				**0.20**			**0.29**			**0.51**		
LL	-5709			-5351								
Number of observations	9081			9081								
Number of parameters	7			41								
Adj. Rho Sq.	0.0918			0.1434								

* significant at 10%

** significant at 5%

*** significant at 1%.

The implied average (in the Dutch society) willingness to sacrifice in order to avoid one fatality directly or indirectly related to COVID-19 equals (rounded to the nearest integer):

- 10 cases of lasting physical injuries;
- 15 cases of lasting mental health problems;
- 18 children with lasting educational disadvantage;
- 77 households with long term decline in net income.

In terms of the relative importance of working pressure in the health care system, we find that a one-step increase in the working pressure corresponds (in terms of disutility to Dutch society) to 3,636 additional fatalities, underscoring the dominant role this variable has been playing in the public debate. In terms of the willingness to accept a higher (one-off) tax to help avoid fatalities, we find that the average Dutch citizen weighs an additional 10,000 fatalities as heavily as a one-off tax increase per household of 2,912 Euro. This implies that Dutch society as a whole (which consists of approximately eight million households) is willing to sacrifice a total additional one-off tax burden of approximately 2.32 million euros.

This ‘value of life’ estimate allows us to indirectly validate our DCE: the Dutch Road Authority [51] employs a value of life metric of 2.61 million Euro in the context of road safety analysis, which is in line with our results. Note that the average life lost in road accidents is likely to be that of a younger person than the average life lost due to COVID-19 directly or indirectly, e.g. due to postponed treatment–this may partially explain that our estimate is lower than the official number used by Dutch authorities. A meta-study done by the OECD [52] reports a median (across studies) value of life of 2.8 million Euros, which also is of the same order of magnitude as our result. As a final indirect validation of our estimates, it is worth pointing out that the factor ten which we obtain between the weight of a fatality versus the weight of a lasting physical injury is about the same as the factor used for Dutch road safety analysis [51].

Before moving to the more sophisticated latent class model, we briefly illustrate how the basic binary logit model can be used to forecast support for policy scenarios. Take the choice between the two policy scenarios depicted in Fig 1. Based on the parameter estimates (and applying Eqs 1 and 2 given in the previous section), the model predicts that 54% of Dutch society would prefer Exit strategy 1, while the remaining 46% would prefer Exit strategy 2. If the government would be able to reduce the impact of strategy 1 on mental health problems (e.g. by means of aggressively increasing funding in mental health care programs), in effect reducing the number of effected individuals from 200 thousand to 20 thousand (i.e., the same number as in strategy 2), the model predicts that support would rise to 70%. Of course, such a mental health program would come with a cost. If the government decides to increase the one-off corona tax in strategy 1 from 1,000 euro to 1,250 euro (which would generate approximately 2 billion euro in taxes) to achieve these mental health benefits, our model predicts that that would still imply a 69% preference level for strategy 1 over strategy 2.

The latent class (LC) model with three classes fits the data significantly better than the binary logit model, even when adjusting for additional parameters; this is to be expected, given that opinions expressed in the public debate on this topic vary widely. The latent class model with four classes did fit the data even better, but we are of the opinion that reasonable class sizes and interpretability are important factors to consider in model selection when the purpose is to generate behavioral insights for policy analysis. We tested a wide range of alternative models and specifications; these are available from the corresponding author upon request. It should be noted, however, that the improvement in fit cannot be entirely attributed to the model describing unobserved heterogeneity, since the LC model also takes the panel structure of the data into account whereas the binary logit model does not. We see that all parameters in all classes are of the expected sign and significant at the 1% level, except for the increase in the number of deaths in Class 1, which is insignificant, and income loss (number of households facing a loss in income) in Class 1, which is only significant at the 5% level.

Looking at the weights obtained per class, the classes can be interpreted as follows: class 1, in which older people are over-represented, is not sensitive to changes in the number of fatalities, but it does care about the other policy-impacts and in particular puts a very high negative weight on tax increases. This finding is interesting and somewhat surprising in light of the fact that individuals in this class are known to be much more likely to die when contracting the virus, compared to younger people. This class contains about 20% of the sample. Class 2, in which higher educated people are over-represented, is highly sensitive to each policy-impact, except for the tax increase (to which they are equally sensitive as the average respondent). This class contains about 29% of our sample. Class 3 (containing about 51% of our sample) is as sensitive as the average respondent to fatality numbers and working pressure in the healthcare sector, while being considerably less sensitive to the other policy effects. In terms of ethical perspectives, Class 2 can be described as a typical consequentialist class, weighing every single impact of the exit strategies. Class 1 weighs most impacts, but not the one which moral psychologists call the sacred attribute (human life), while Class 3 can be considered to combine the consequentialist and deontological perspectives as it weighs all effects, but puts less (compared to the average respondent) weight on effects other than the increase in fatalities and the working pressure in the healthcare system.

In Table 4, we show the sample level willingness-to-sacrifice estimates for each sub-group (segment) of respondents implied by our class probability functions, as well as the associated 95% confidence intervals. Standard errors were calculated using the Delta method [53]. In terms of willingness to accept an increase in the number of people with lasting physical injuries to avoid one fatality directly or indirectly related to COVID-19, we find several differences between subgroups of respondents. For example, highly educated women between 66 and 74 years old are only willing to accept an increase of 10 people with lasting physical injuries for each avoided fatality, whereas men between 18 and 25 with low education are willing to accept an increase of more than 16 people with lasting physical injuries for each avoided fatality. Looking at the other policy dimensions, we see the same general trend: older and more highly educated people in general, and women in particular, are willing to sacrifice (per fatality avoided) fewer people with mental injuries, fewer children at an educational disadvantage and fewer households with an income loss, compared to younger people with lower education, and younger men in particular. In many cases the differences in willingness to sacrifice between segments of the population are large and significant judging by the non-overlapping confidence intervals.

**Table 4:** Sample level unconditional estimates of willingness-to-sacrifice-a-fatality for a reduction in physical injuries, mental injuries, educational disadvantage, and income loss; segmented by gender, age and education level.

Description	Physical injuries	95% CI	Mental injuries	95% CI	Educational disadvantage	95% CI	Income loss	95% CI
**Men aged 18–25 with low education**	16.13	(15.7, 16.57)	25.83	(24.92, 26.73)	52.06	(49.08, 55.04)	131.22	(130.8, 131.65)
**Men aged 18–25 with medium education**	16.09	(15.68, 16.5)	25.66	(24.78, 26.53)	51.69	(48.79, 54.59)	130.61	(130.2, 131.02)
**Men aged 18–25 with high education**	14.59	(14.25, 14.93)	22.62	(21.9, 23.33)	44.19	(41.88, 46.49)	116.21	(115.87, 116.55)
**Men aged 26–35 with low education**	12.76	(12.35, 13.16)	22.09	(21.22, 22.96)	40.67	(38.26, 43.09)	106.07	(105.7, 106.44)
**Men aged 26–35 with medium education**	13.14	(12.77, 13.51)	22.44	(21.62, 23.26)	41.83	(39.46, 44.19)	108.74	(108.39, 109.1)
**Men aged 26–35 with high education**	12.16	(11.86, 12.47)	20.35	(19.65, 21.04)	36.74	(34.81, 38.67)	99.1	(98.81, 99.4)
**Men aged 36–45 with low education**	13.46	(13.07, 13.85)	22.49	(21.67, 23.32)	42.39	(39.94, 44.84)	110.45	(110.09, 110.81)
**Men aged 36–45 with medium education**	13.69	(13.33, 14.05)	22.61	(21.84, 23.39)	42.92	(40.54, 45.3)	111.85	(111.5, 112.19)
**Men aged 36–45 with high education**	12.38	(12.09, 12.66)	19.75	(19.13, 20.37)	35.99	(34.16, 37.83)	98.77	(98.5, 99.05)
**Men aged 46–55 with low education**	11.39	(11.03, 11.75)	20.16	(19.31, 21.01)	35.33	(33.3, 37.35)	94.91	(94.59, 95.24)
**Men aged 46–55 with medium education**	11.8	(11.48, 12.12)	20.49	(19.7, 21.28)	36.5	(34.52, 38.47)	97.69	(97.38, 98)
**Men aged 46–55 with high education**	10.84	(10.58, 11.1)	18.19	(17.53, 18.84)	31.05	(29.51, 32.6)	87.63	(87.38, 87.88)
**Men aged 56–65 with low education**	10.96	(10.64, 11.28)	19.22	(18.43, 20.02)	33.07	(31.26, 34.88)	90.65	(90.35, 90.95)
**Men aged 56–65 with medium education**	11.33	(11.04, 11.62)	19.45	(18.72, 20.17)	33.98	(32.23, 35.73)	92.95	(92.67, 93.23)
**Men aged 56–65 with high education**	10.32	(10.09, 10.54)	16.83	(16.26, 17.41)	27.91	(26.62, 29.21)	81.93	(81.71, 82.15)
**Men aged 66–74 with low education**	11.04	(10.75, 11.33)	18.72	(18.02, 19.43)	32.29	(30.65, 33.93)	89.87	(89.59, 90.15)
**Men aged 66–74 with medium education**	11.31	(11.04, 11.58)	18.78	(18.13, 19.42)	32.76	(31.18, 34.35)	91.30	(91.03, 91.57)
**Men aged 66–74 with high education**	10.16	(9.96, 10.36)	15.76	(15.28, 16.25)	25.80	(24.7, 26.91)	78.68	(78.48, 78.88)
**Men aged 75 and older with low education**	12.94	(12.59, 13.29)	21.52	(20.8, 22.25)	39.93	(37.81, 42.04)	105.64	(105.31, 105.97)
**Men aged 75 and older with medium education**	13.15	(12.81, 13.49)	21.57	(20.87, 22.27)	40.31	(38.23, 42.38)	106.76	(106.43, 107.08)
**Men aged 75 and older with high education**	11.74	(11.46, 12.02)	18.41	(17.85, 18.97)	32.71	(31.14, 34.29)	92.53	(92.27, 92.8)
**Women aged 18–25 with low education**	15.74	(15.34, 16.15)	24.88	(24.03, 25.74)	49.83	(47.05, 52.62)	127.13	(126.73, 127.53)
**Women aged 18–25 with medium education**	15.62	(15.25, 16)	24.58	(23.77, 25.39)	49.13	(46.46, 51.8)	125.84	(125.45, 126.22)
**Women aged 18–25 with high education**	13.77	(13.48, 14.07)	20.83	(20.2, 21.45)	39.87	(37.87, 41.86)	108.07	(107.77, 108.37)
**Women aged 26–35 with low education**	13.30	(12.92, 13.67)	22.33	(21.53, 23.13)	41.87	(39.48, 44.25)	109.27	(108.91, 109.62)
**Women aged 26–35 with medium education**	13.55	(13.2, 13.89)	22.48	(21.72, 23.24)	42.48	(40.16, 44.81)	110.84	(110.5, 111.18)
**Women aged 26–35 with high education**	12.28	(12.01, 12.55)	19.69	(19.09, 20.29)	35.75	(33.96, 37.54)	98.14	(97.88, 98.41)
**Women aged 36–45 with low education**	13.62	(13.26, 13.97)	22.10	(21.35, 22.86)	41.91	(39.57, 44.25)	110.29	(109.94, 110.63)
**Women aged 36–45 with medium education**	13.71	(13.38, 14.03)	22.01	(21.29, 22.72)	41.86	(39.62, 44.11)	110.49	(110.17, 110.82)
**Women aged 36–45 with high education**	12.08	(11.83, 12.32)	18.46	(17.93, 18.98)	33.27	(31.66, 34.89)	94.28	(94.04, 94.52)
**Women aged 46–55 with low education**	11.98	(11.65, 12.31)	20.30	(19.55, 21.05)	36.41	(34.41, 38.41)	98.12	(97.81, 98.43)
**Women aged 46–55 with medium education**	12.25	(11.96, 12.55)	20.41	(19.72, 21.1)	37.00	(35.08, 38.91)	99.71	(99.42, 100)
**Women aged 46–55 with high education**	11.00	(10.78, 11.23)	17.42	(16.89, 17.95)	29.92	(28.52, 31.33)	86.64	(86.41, 86.86)
**Women aged 56–65 with low education**	11.43	(11.14, 11.72)	19.05	(18.37, 19.74)	33.43	(31.67, 35.18)	92.54	(92.26, 92.82)
**Women aged 56–65 with medium education**	11.65	(11.38, 11.91)	19.04	(18.42, 19.66)	33.71	(32.04, 35.38)	93.57	(93.31, 93.83)
**Women aged 56–65 with high education**	10.38	(10.18, 10.57)	15.84	(15.38, 16.3)	26.24	(25.08, 27.39)	79.90	(79.7, 80.09)
**Women aged 66–74 with low education**	11.27	(11.01, 11.53)	18.10	(17.5, 18.69)	31.50	(29.96, 33.04)	89.51	(89.25, 89.77)
**Women aged 66–74 with medium education**	11.38	(11.14, 11.63)	17.92	(17.37, 18.48)	31.35	(29.87, 32.83)	89.66	(89.41, 89.91)
**Women aged 66–74 with high education**	10.04	(9.86, 10.22)	14.57	(14.18, 14.97)	23.52	(22.54, 24.5)	75.30	(75.13, 75.48)
**Women aged 75 and older with low education**	13.01	(12.68, 13.34)	20.91	(20.24, 21.57)	38.94	(36.93, 40.94)	104.55	(104.24, 104.87)
**Women aged 75 and older with medium education**	13.07	(12.75, 13.39)	20.73	(20.08, 21.38)	38.71	(36.76, 40.66)	104.43	(104.12, 104.74)
**Women aged 75 and older with high education**	11.39	(11.14, 11.65)	17.00	(16.49, 17.52)	29.73	(28.3, 31.17)	87.56	(87.31, 87.81)
**Mean**	**12.47**		**20.28**		**37.06**		**100.46**	
**Min**	**10.04**		**14.57**		**23.52**		**75.30**	
**Max**	**16.13**		**25.83**		**52.06**		**131.22**	

Returning to the fact that our sample, while representative in terms of gender and age, is somewhat skewed towards highly educated people (see Table 2), these segment-specific estimation results imply that our aggregate level estimates for society’s weighing of various policy impacts of exit strategies is likely to somewhat underestimate the weight attached to fatalities and to somewhat overestimate the weight attached to other policy impacts such as physical and mental injuries, educational advantage, and income loss. More generally, these results suggest that segmentation along socio-demographic lines is needed to obtain a nuanced view of society’s preferences related to COVID-19 policies.

With a view to exploring potential causes behind differences in weights attached to fatalities and other policy impacts, we estimated a series of two-class latent class models, where class membership was determined by respondents’ perceived risk that they or a relative would contract COVID-19, would become severely ill if having contracted the virus, would be hospitalized if contracting the virus, or would die if contracting the virus. None of these variables, which were all measured using five-point Likert-scales, turned out to have a significant effect on class membership (and hence, on respondents’ weighing of the policy impacts). We also tested a model where class membership was a function of whether or not one or more of a respondent’ relatives had contracted the virus; 58 respondents indicated that this was the case. (note: only two respondents indicated that they had contracted the virus themselves, this number being too low to use in our analyses) Using this variable as a predictor of class membership, Table 5 shows that respondents whose relative(s) had been infected by the virus were significantly less likely to belong to Class 1 which does not put a significant weight on fatalities. This is in line with intuition.

**Table 5:** Results from a latent class model with two classes where class membership is determined based on whether you have a relative who has tested positive for COVID-19.

	Class 1			Class 2		
Policy impacts	Est		S.e.	Est		S.e.
Deaths (x10,000)	-0.2808		0.2477	-0.6066	***	0.0388
Physical injuries (x100,000)	-1.1843	***	0.1679	-0.5531	***	0.0272
Mental injuries (x100,000)	-0.2245	**	0.1003	-0.4268	***	0.0219
Educational disadvantage (x100,000)	-0.5248	***	0.0748	-0.295	***	0.0167
Income loss (x1,000,000)	-1.039	**	0.4314	-0.7511	***	0.0447
Tax increase (x1,000 euro)	-1.0174	***	0.0891	-0.1001	***	0.0104
Increase in WP	-0.5107	***	0.1299	-0.1872	***	0.0138
**Class probability functions**						
Constant	-1.4257	***	0.1158	0		(fixed, ref)
No relative infected	0.0000		(fixed, ref)	0		(fixed, ref)
A relative infected	-0.9885	***	0.5272	0		(fixed, ref)
**Average class probability**	0.1870			0.8126		
LL	-5441					
N	9036					
K	16					
Adj. Rho Sq.	0.129					

* significant at 10%

** significant at 5%

*** significant at 1%.

Our results for taboo trade-off aversion (see Table 6 for the full estimation results) can be summarized as follows: in a three class model where we allowed all parameters, including the taboo trade off aversion parameter, to vary between classes, we find two classes with a significant and negative taboo-penalty associated with policies that involve (simultaneously) a lower tax and a higher number of fatalities than the alternative policy. For one of these classes, containing 21% of our sample, the taboo penalty is large (-1.44) whereas in the other class, containing 54% of our sample, the taboo penalty is of moderate size (-0.42). A third class (containing a minority of 25% of our sample), surprisingly features a large and positive taboo parameter (2.35), implying that individuals in this class actually favor the combination of lower taxes and higher fatality rates.

**Table 6:** Estimation results for the taboo trade-off latent class model with three classes.

	Class 1			Class 2			Class 3		
Utility function	Est		S.e.	Est		S.e.	Est		S.e.
Deaths (x1,000)	0.3606		0.4069	-2.3775	***	0.7079	-0.3617	***	0.1169
Physical injuries (x10,000)	-1.4584	***	0.1967	-1.0712	***	0.1104	-0.3884	***	0.0536
Mental injuries (x10,000)	-0.3308	***	0.1000	-1.3759	***	0.2467	-0.2002	***	0.0381
Educational disadvantage (x10,000)	-0.5652	***	0.0671	-0.8658	***	0.0921	-0.1421	***	0.0349
Income loss (x100,000)	-0.9984	**	0.3406	-1.9064	***	0.2194	-0.4669	***	0.0751
Tax increase (x1,000 euro)	-1.1407	***	0.1379	0.0716	***	0.1324	-0.1244	***	0.0315
Increase in WP	-0.3870	***	0.0795	-0.2065	***	0.0469	-0.2139	***	0.0193
**Taboo**	**-1.4388**	***	**0.6993**	**2.3497**	***	**1.0961**	**-0.4195**	***	**0.1921**
**Class probability functions**									
Constant	-0.9535	***	0.1397	-0.7497	***	0.2288	0		(fixed, ref)
**Average class probability**	**0.21**			**0.25**			**0.54**		
LL	-5375								
N	9081								
K	26								
Adj. Rho Sq.	0.1419								

* significant at 10%

** significant at 5%

*** significant at 1%.

Some caution is in place though, when interpreting these taboo aversion-related outcomes: correlations between the taboo penalty-parameters on the one hand, and the tax- and fatality-related parameters on the other hand, were quite high (>0.80). This indicates, that the model struggles to disentangle the direct effects of the tax- and fatality-variables on the one hand, and their joint indirect effect through the taboo-dummy variable on the other hand. This is not surprising, given that the experiment was not designed specifically to pick up these two separate but subtly related effects in an econometrically efficient way. We performed additional analysis to verify our results, including a three class model where the taboo parameter was allowed to vary across classes while the attribute weights were constrained to be the same across classes, as well as a three class model where the taboo parameter was allowed to vary across classes while the attribute weights were fixed to their binary logit sample-level estimates. Estimation results for these models (which can be obtained from the corresponding author) showed that, as expected, they had a much lower model fit than the original taboo trade off aversion model which allowed the taboo parameter and the attribute weights to vary across classes, but they avoid high correlations between parameters. These models identified a taboo aversion for only about 10% of the sample. This indicates that more research is needed to draw definite conclusions about the role of taboo trade off aversion in the context of lockdown relaxation policies.

## 5. Conclusions and discussion

This paper presented the results of an empirical study into Dutch society’s preferences of COVID-19 related government policies, specifically in terms of the weights attached to various impact-dimensions of such policies. At the aggregate level–i.e., combining choices made by all respondents–as well as for particular segments of the population, we obtain estimates for society’s willingness to accept or sacrifice fatalities in order to avoid physical, mental, educational, and economic impacts of lock-downs. The fact that the implied ‘value of life’ estimate obtained on our sample is close to the value that is used by the Dutch government in other contexts, lends credibility to our results. What is perhaps the most striking finding of our study, is the large heterogeneity within the population in terms of the weights attached to various policy impacts: first, while some groups appear to weigh all impacts in line with what would be prescribed by consequentialist ethics, other groups appear to put much weight on some impacts while ignoring other impacts. Second, while a majority dislikes policies that imply a taboo trade-off between lives and taxes, a sizeable minority in fact favors such policies. Third, also within each group, there appears to be a substantial variation in terms of the weights attached to various policy impacts. Some of this heterogeneity can be traced back to whether one has a relative that has contracted the virus (this leads to a higher weight on avoiding fatalities), but classical sociodemographic differences (gender, age, education level) appear to play an important role as well. This high level of preference heterogeneity should come as no surprise to those who have been following the heated debates about COVID-19 policies in various countries including the Netherlands; this result is also in line with the high levels of heterogeneity found in several other COVID-19 choice experiments and surveys (see papers cited in the Introduction).

In the face of the COVID-19 crisis, policy makers worldwide need to make choices with far-reaching consequences, based on a wide variety of considerations and limited by a high degree of uncertainty. The results of our research are no more than a small piece of this immense puzzle, and are subject to a number of limitations: first, while great care was exercised to develop a choice experiment aimed at obtaining trustworthy responses, it must be acknowledged that the choices made by respondents were made between hypothetical policies. As such, care must be exercised when interpreting and applying our estimation results. Second, although our sample was representative in terms of gender and age, and despite having a high response rate among members of a representative panel, we did obtain an over-representation of highly educated people. Combining this notion with our estimation results, this implies that our aggregate level results are slightly overestimating society’s willingness to accept a fatality in order to reduce negative impact on educational disadvantage, economic impacts and other policy impacts. Third, our data were collected at a specific point in time in a specific geographical and cultural context, which in itself limits the generic applicability of our results. Notwithstanding these limitations, we believe that our results can be useful in four ways in developing policies with regard to a possible (further) relaxation of the intelligent lockdown in the Netherlands as well as in other countries.

First, upon inspection of our estimation results, including the Latent Class models, we find that most of our respondents appear to have an eye for both the direct health-related effects of relaxation policies and their indirect impacts on mental health, the economy, education and their personal financial situation. Although health impacts are of great importance to our respondents, most seem to employ a consequentialist ethical perspective by balancing various dimensions of policies. This suggests that an open view of policy makers, taking into account the broad range of effects of policies, would be appreciated by citizens. Nonetheless, our result that about three quarters of our sample have a moderate to high aversion against making ‘taboo tradeoffs’, suggest that governments need to be careful in their decision-making and in how policies and their effects are communicated to the public.

Secondly, our results enable policy makers to determine whether the net valuation in society is positive or negative for particular combinations of policy effects. Take, for example, the situation in which a certain policy measure leads to an expected reduction in deaths, but also to an increase in the number of people who will suffer from lasting mental health problems such as depression. Our results indicate that, if the number of people who experience such problems as a result of the policy is fewer than about fifteen per avoided death, the policy is assessed positively by the average Dutch person. However, if the number is higher, the net valuation is negative.

Thirdly, our results enable policy makers to identify levels of support and opposition among Dutch citizens for different policy packages. For example, the binary logit model, fed by the estimated parameters, can be used to determine the percentage of Dutch people who support a certain policy package over another one, as was illustrated in Section 4. For this, the effects of the policy package must of course be within the scope of those presented in Table 1.

And fourthly, our model can be used to determine whether the average adult citizen prefers a proposed policy package over a particular reference case. This reference case can be “a continuation of current policy” or “no restrictive measures”. For this, it is important that the reference case can be specified in terms of the impacts on (a selection of) the policy impacts included in our experiment.

In terms of avenues for further research, we believe that assessing the spatial and temporal transferability of our results is important, before conclusions for other countries, and for future situations, can be derived. Regarding geography, given the considerable differences across countries in terms of culture, COVID-19 policies and the effects of the virus on society, we expect that weights for various policy effects will differ across countries. Moreover, some effects included in our experiment might be less relevant in other countries, while factors excluded in our study may be highly relevant in different geographical contexts. Regarding timing, we expect that as the situation changes in terms of the threat of the virus and the visibility of e.g. economic effects, societal preferences and trade-offs will change, too. It would be interesting to test for this, by repeating our experiment in due time. Nonetheless, we feel that having these timely estimates available helps (local) policy makers in the short term. A final suggested avenue for further research relates to the hypothetical nature of our experiment: if, as seems likely, one or more countries will in due time ask their citizens to vote for particular COVID-19 policies in a referendum style voting process, such ‘real’ data could be used to make a comparison with data obtained by means of hypothetical choice experiments such as ours; see Hainmueller et al. [31] for an example of such a comparison. In general, we hope that future study efforts aimed at measuring citizens’ tradeoffs and preferences concerning the policy impacts of lockdowns in different countries (triggered by COVID-19 or another pandemic) can use our study as a stepping stone.

## Supporting information

S1 Appendix Description of the policy impacts. (DOCX)
