# Supplementary material for: Diabolical dilemmas of COVID-19: An empirical study into Dutch society’s trade-offs between health impacts and other effects of the lockdown
Source: PLoS One. 2020 Sep 16;15(9):e0238683. doi: 10.1371/journal.pone.0238683 (PMC7494093; doi:10.1371/journal.pone.0238683)
Supplement: S1 Appendix — (DOCX) [file pone.0238683.s001.docx]

**Appendix: Description of the policy impacts**

Below we present the full explanation of the seven policy impacts as presented to the participants of our discrete choice experiments.

Increase in the number of deaths

Due to the outbreak of the coronavirus the number of Dutch people who die will be higher in the period 1 May 2020 - 1 January 2021 than in a state of affairs without an outbreak of the coronavirus in the Netherlands. The number of deaths increases due to the coronavirus, or because treatments for a condition other than corona should be postponed. We assume that the actual number of deaths caused by the coronavirus will be higher than the number of deaths reported by the Dutch National Institute for the Public Health. The increase in the number of deaths will depend on the exit strategy selected by the government.

Number of people with lasting physical health problems

Due to the outbreak of the coronavirus the number of Dutch people who suffer from lasting physical injuries will be higher in the period 1 May 2020 - 1 January 2021 than in a state of affairs without an outbreak of the coronavirus in the Netherlands. For example, infection with the coronavirus can lead to lasting damage to the lungs and other organs. The number of people with lasting physical problems may also increase because treatments for conditions other than corona have to be postponed. This research is only about the increase of permanent physical complaints and not about temporary physical complaints. The increase in lasting physical injuries will depend on the exit strategy selected by the government.

Number of people with lasting mental health problems

Due to the outbreak of the coronavirus the number of Dutch people who suffer from lasting mental injuries will be higher in the period 1 May 2020 - 1 January 2021 than in a state of affairs without an outbreak of the coronavirus in the Netherlands. Relatives of people who die from corona can face lasting mental injuries (because they have to say goodbye in a different way), but lasting mental injuries can also arise as a result of loneliness, depression, burnout complaints or domestic violence. This research is only about the increase in lasting mental injuries and not about the temporary psychological complaints. The increase in lasting mental injuries will depend on the exit strategy selected by the government.

Additional number of children who experience lasting educational disadvantage when leaving secondary school

Due to the outbreak of the coronavirus the number of children who are left with a permanent educational disadvantage will be higher in the period 1 May 2020 - 1 January 2021 than in a state of affairs without an outbreak of the coronavirus in the Netherlands. With a lasting educational disadvantage we mean that a child leaves secondary school later than in a situation without the corona crisis or that a child obtains a lower diploma. Hence, we exclude the children who face a short-term educational disadvantage, but who will catch up before leaving secondary school. For many preschoolers, for example, it will apply that they can catch up in a few years. To be clear, we consider the number of children who no longer make up for the educational disadvantage after the corona crisis. The increase in the number of children who face lasting educational disadvantage will depend on the exit strategy selected by the government.

Number of households that suffer a long-term decline in net income of at least 15%

The Dutch government supports the economy with a large financial support package. Yet, as a result of the corona crisis, people will lose their jobs in the period May 1, 2020 - January 1, 2021 and companies will make less profit or even go bankrupt. The increase in the number of households that will suffer a decline in net income of at least 15% for a long time (at least three years long) will depend on the exit strategy selected by the government.

One-off increase in taxes

The support packages cost a lot of money. Imagine that in the future part of these costs will be covered through taxation. In this study, we look at a one-off tax on January 1st 2023. The one-off tax that we will present is an average. Households with a high income will have to pay a higher amount than households with a lower income. The one-off corona tax may differ between the exit strategies.

Work pressure in the healthcare sector

The exit strategies differ in the workload for people who work in the health care sector (think of nurses and doctors, but also cleaners and laboratory employees). In this study, four options are possible: 1) The workload in the healthcare sector in the period 1 May 2020 - 1 January 2021 remains the same as in the current situation; 2) The workload is considerably higher than in the current situation; 3) The workload drops considerably to a level that lies between the current situation and the period before the corona crisis (early February 2020); 4) The workload even drops back to the level before the corona crisis (February 2020).
